# *Staphylococcus aureus* Small Colony Variants (SCVs): a road map for the metabolic pathways involved in persistent infections

**DOI:** 10.3389/fcimb.2014.00099

**Published:** 2014-07-28

**Authors:** Richard A. Proctor, André Kriegeskorte, Barbara C. Kahl, Karsten Becker, Bettina Löffler, Georg Peters

**Affiliations:** ^1^Departments of Medical Microbiology/Immunology and Medicine, University of Wisconsin School of Medicine and Public HealthMadison, WI, USA; ^2^Institute of Medical Microbiology, University Hospital, MünsterMünster, Germany

**Keywords:** *Staphylococcus aureus*, small colony variants, metabolism, RNA processing, post-transcriptional, persistence

## Abstract

Persistent and relapsing infections, despite apparently adequate antibiotic therapy, occur frequently with many pathogens, but it is an especially prominent problem with *Staphylococcus aureus* infections. For the purposes of this review, persistence will encompass both of the concepts of long term survival within the host, including colonization, and the concept of resisting antibiotic therapy even when susceptible in the clinical microbiology laboratory. Over the past two decades, the mechanisms whereby bacteria achieve persistence are slowly being unraveled. *S. aureus* small colony variants (SCVs) are linked to chronic, recurrent, and antibiotic-resistant infections, and the study of SCVs has contributed significantly to understanding of persistence. In our earlier work, defects in electron transport and thymidylate biosynthesis were linked to the development of the SCV phenotype (reviewed in 2006), thus this work will be discussed only briefly. Since 2006, it has been found that persistent organisms including SCVs are part of the normal life cycle of bacteria, and often they arise in response to harsh conditions, e.g., antibiotics, starvation, host cationic peptides. Many of the changes found in these early SCVs have provided a map for the discovery mechanisms (pathways) for the development of persistent organisms. For example, changes in RNA processing, stringent response, toxin-antitoxin, ribosome protein L6 (RplF), and cold shock protein B (CspB) found in SCVs are also found in other persisters. In addition, many classic persister organisms also show slow growth, hence SCVs. Recent work on *S. aureus USA300* has elucidated the impact of aerobic expression of arginine deiminase genes on its ability to chronically colonize the skin and survive in abscesses. *S. aureus* SCVs also express arginine deiminase genes aerobically as well. Thus, many pathways found activated in electron transport type of SCVs are also increased in persisters that have intact electron transport. Many of these changes in metabolism result in slow growth; hence, small colonies are formed. Another common theme is that slow growth is also associated with reduced expression of virulence factors and enhanced uptake/survival within host cells. These adaptations to survive within the host are rooted in responses that were required for organisms to survive in a harsh environment long before they were mammals on the earth.

## Introduction

Persistent and relapsing infections, despite organism susceptibility and apparently adequate antibiotic therapy, occur frequently with many pathogens, but it is an especially prominent problem with *Staphylococcus aureus* infections (Lowy, 1998). The basis for persistence has been slowly unraveled over the past two decades, and many of the pathways involve changes in metabolism. One phenotype of microorganisms has helped to pave the way to understand persistence, and this is represented by small colony variants (SCVs) (Proctor et al., 2006). The linkage between *S. aureus* SCVs and persistent infection was first reported in a small clinical series in 1995 (Proctor et al., 1995). Because *S. aureus* SCVs were able to establish an intracellular infection in cultured cells, it was hypothesized that this might form the basis for the development of persistent infections (Balwit et al., 1994). Moreover, the instability of these SCVs wherein they could revert to the parental normal phenotype would also provide a mechanism for relapsing, virulent infections. An important part of the ability to persist was associated with the quiescent metabolic state (Proctor et al., 1995). *S. aureus* SCVs were found to produce fewer lytic enzymes, thereby allowing them to persist within the host cells (Proctor et al., 2006). Since then, further work has demonstrated enhanced uptake by *S. aureus* SCVs due to a high expression of adhesins that facilitate host cell uptake (Sendi and Proctor, 2009; Tuchscherr et al., 2010). Early studies suggested that the intracellular milieu could select for SCVs (Vesga et al., 1996), perhaps because cationic antimicrobial peptides, e.g., lactoferrin, provide the selective pressure within host cells (Samuelsen et al., 2005; Gläser et al., 2014). While early studies suggested that the intracellular milieu could select for SCVs (Vesga et al., 1996), more recent and detailed studies demonstrated that a high percentage of the initial inoculum (as much as 25%) of *S. aureus* could undergo phenotypic switching to produce SCVs (Tuchscherr et al., 2011). This was shown to not only occur in tissue culture, but also within the kidneys and bones of these intravenously challenged mice (Horst et al., 2012). The SCVs that persisted stimulated a reduced immune response, expressed increased adhesins, and reduced toxins (Tuchscherr et al., 2010). Moreover, the failure of *S. aureus* SCVs to stimulate host cells to produce hypoxia-inducible factor, which would normally alert the host to the presence of intracellular pathogens, also is important for promoting persistence (Werth et al., 2010). Finally, persistence of *S. aureus* SCVs may also relate to the relative inefficiency of antibiotics to clear the organisms with host cells (Garcia et al., 2013). Thus, *S. aureus* SCVs have been established as intracellular pathogens that persist within the host. This review will largely concentrate upon information that has been published since the 2006 review of SCVs (Proctor et al., 2006) and will emphasize metabolic mechanisms involved in persistence. *S. aureus* SCVs as a model persister organism

How do *S, aureus* SCVs relate to the broader concept of persistence? Many other species of bacteria that persist within the host also form SCVs (Proctor et al., 2006). Hence, the specific example of *S. aureus* SCVs is also found within many other pathogenic species. In addition, persisters share many characteristics, and these are also part of the SCV life cycle. Persisters and *S. aureus* SCVs grow slowly due to decreased metabolic activity (Amato et al., 2014). A subset of organisms within many bacterial populations can be part of the normal growth cycle (Lechner et al., 2012), and this is certainly true for *S. aureus* SCVs, which are part of the normal growth cycle that occurs without any external stresses (Massey et al., 2001; Edwards, 2012). However, persisters also form as a response to harsh conditions such as antibiotics, acid, and starvation stresses (Morikawa et al., 2010). Biofilm formation can be thought of as a persister strategy (Abdallah et al., 2014). Naturally, persistence is inter-related to chronic colonization and chronic infections as these may be considered types of persistence.

## Electron transport type of *S. aureus* SCVs

The initial description of *S. aureus* SCV persisters identified alterations in electron transport due to mutations in hemin and menadione biosynthetic genes, which resulted in a loss of menaquinone and the heme prosthetic group in cytochromes (Proctor et al., 2006). Others found that mutations in the cytochrome c assembly protein (CtaA) also produced SCVs (Clements et al., 1999). Thymidine auxotrophic SCVs were soon added (Proctor et al., 2006) to the list of metabolic changes in SCVs. Mutations in *thyA* were found to produce SCVs that were phenotypically very similar to the electron transport deficient (Chatterjee et al., 2008). The reason that thymidine-dependent SCVs behaved like electron transport defective organisms came through decreased Krebs cycle activity (Chatterjee et al., 2005, 2007). Of course, when Krebs cycle is decreased, electron transport is down regulated. It was found that *thyA* mutation was associated with decreased aconitase (CitB) abundance. A further connection became apparent wherein CitB expression requires ClpC, which is reduced in *thyA* mutants (Chatterjee et al., 2005, 2007, 2008). Hence, *thyA* mutants are grouped with organisms defective in electron transport. All of the electron transport types of SCVs are able to persist within the host and/or cultured mammalian cells.

## Changes in RNAIII metabolism in electron transport group of SCVs

As noted above, slow growth is found in many, but not all, organisms that persist. When examining clinical and tissue-cultured induced *S. aureus* SCVs, only ~20% can be assigned to a defined auxotrophy (Vesga et al., 1996; Proctor et al., 2006; Tuchscherr et al., 2011). Therefore, the underlying mechanism for forming a persistent SCV is unknown. Publications in the past six years have revealed a number of mutations that result in SCVs, but most of these have not been examined in clinical SCVs. Surprisingly, many of these newer mutations can be grouped as being involved with the ribosome and RNA processing. This will be discussed in the balance of this article.

One of the first indications that electron transport type of *S. aureus* SCVs were atypical for RNA processing came from the observation that there was a complete absence of RNAIII (Vaudaux et al., 2002; Kohler et al., 2003, 2008; Kahl et al., 2005), which is the effector molecule in the quorum sensing apparatus, *agr* (accessory gene regulator), in *S. aureus* (Novick and Geisinger, 2008). RNAIII arises from nontranslated portion of the *hld* (hemolysin D) gene in the *agr* operon. RNAIII positively regulates production of toxins and proteases but negatively regulates adhesins.

Because *agr* is a quorum-sensing system, one might postulate that the decreased growth of electron-transport types of SCVs simply do not reach sufficient density to activate the quorum-sensing pathway. This seems unlikely for several reasons. The first is that SCV colonies on blood agar, wherein the local density is very high, fail to produce hemolytic colonies, and the hemolysins are controlled by *agr*/RNAIII (Proctor et al., 2006). The second is that RNAIII is not found in any detectable amount in SCVs (Vaudaux et al., 2002; Kohler et al., 2003, 2008; Kahl et al., 2005), which is distinctly unusual in that a basal level of RNAIII is produced in *S. aureus* during all phases of growth (Novick and Geisinger, 2008). Surprisingly, mutation in *citB* produces a major growth defect, yet a 14-fold increase in RNAIII was found in the *citB* mutant as compared to the parent strain *S. aureus* strain. This work was performed in a clinical isolate, SA564, in TSB supplemented with 0.25% glucose (Somerville et al., 2002). The differences in RNAIII production were found in the post-exponential phase of growth, with no differences in the exponential phase of growth. Finally, RNAIII is reduced under anaerobic conditions in *S. aureus* MN8 along with the production of toxic shock syndrome toxin–1 (TSST-1) (Pragman et al., 2004), which is regulated by RNAIII. However, the anaerobic suppression of TSST-1 production can be reversed if excess quantities of pyruvate are added to the growth medium, resulting in TSST-1 production during late stationary phase (Table 1). This shows that the anaerobic conditions alone do not block RNAIII-regulated production of TSST-1, which is consistent with an earlier report where both pyruvate and uracil were added to the culture medium (Sarafian and Morse, 1987). Taken together, these data strongly suggest an altered RNAIII metabolism when electron transport is reduced, which is not simply due to reduced growth/concentration of the quorum-sensing peptide.

**Table 1:** **Anaerobic production of toxic shock syndrome toxin – 1**.

**mM Pyruvate added**	**ng TSST-1/10^6^ cfu**
0	0.7
40	0.05
200	24.6
400	63

Exogenous pyruvate and anaerobic toxin production in *S. aureus* MN8 grown in tryptic soy broth for 18 h under anaerobic conditions, and supernatants collected for TSST-1 measurements. TSST-1 was measured by an ELISA as previously described (McNamara et al., 2009).

There is a strong inverse connection between *agr*, hence RNAIII, and persistent infection. The loss of the major virulence factor regulator in *S. aureus*, *agr*, has been associated with chronic infections. These *agr* mutants have been obtained from clinical isolates from patients with long-term lung infections (cystic fibrosis) (Kahl et al., 2005; Hirschhausen et al., 2013), chronic catheter infections (Rothfork et al., 2003; Yarwood and Schlievert, 2003), and chronic soft tissue infections (Schwan et al., 2003; Beenken et al., 2004). Of interest, the development of skin abscesses requires the presence of *agr* to establish the initial infection, but persistence within the abscess is associated with decreased RNAIII (Wright et al., 2005). Thus, these infections are strongly linked to situations wherein the organism persists within the host and resists antibiotic therapy. This suggests that a complete absence of the RNAIII effector molecule from the *agr* operon is associated with persistence.

## The RNA degrasome and SCVs

Of interest, small colonies and changes in RNA metabolism are seen in mutants in *cshA, cspB, rsaE, rplF, mazF*, and *rsh* (see Table 2). The role of these mutations in persistent infection is discussed in the balance of this manuscript.

**Table 2:** **Comparison of phenotypes in SCVs and persisters**.

**Phenotype**	**SCV (*men/hem/ctaA*) Also, unsatu-rated fatty acids**	**SCV (*thyA*)**	**Δ *cshA***	**Δ *cspB***	**RsaE over production**	***rplF* mutant (Ribosome protein L6)**	**Increased MazF (Toxin – AT)**	**Rsh constitutively active(RelA)**
**Biochemical**	**Electron transport**	**Thymidy-late synthesis**	**Hellicase (ATP dependent)**	**Cold shock protein**	**Regulatory small RNA**	**Fusidic acid resistance**	**Endoribo-nuclease**	**ppGpp synthesis**
Colony size	Small	Small	Small(22°C)	Small	Small	Small	Small	Small
Persistence	Yes	Yes					Yes	Yes (↑PSMs)
Hla	Decrease	Decrease	Increase				Decreased	Increase
Hld	Decrease	Decrease	Increase					Increase
Spa	Decrease	Increase	Decrease				Decrease	Decrease
FnBP	Increase	Increase						Increase
RNAIII	Decrease	Decrease	Increase		Decreased			Increase(↓RnpA)
CPS	Increase	Decrease	Increase					Increase
Biofilm	Increase	Increase	Decrease					Increase capsule
TCA	Decrease	Decrease	Decrease		↓*sucD/*TCA			Increase
ClpC/ClpP		Decreased	Decrease					Decrease
Ilv pathway	Increase							Increase
ppGpp								Increase
Cell wall thick	Increased	Increased						
Pigment	Decreased	Decreased		Decreased		Decreased	Decreased	
Cold growth	Increased CspB		Decreased	Decreased				
Antibiotic	AG^r^, β L^r^, Dapto^s^	SXT^r^,				AG^r^		

AG^r^, aminoglycoside resistant; β L^r^, betalactam resistant; Dapto^s^, daptomycin susceptible; ClpC, ATP-dependent protease C; ClpP, ATP-dependent protease P; CSP, capsular polysaccharide; CspB, cold shock protein B; FnBP, fibronectin binding protein; Hla, α-toxin; Hld, δ-toxin; Ilv, Isoleucin, leucine, valine; Spa, Protein A; SXT^r,^, sulfamethoxazole-trimethoprim resistant; sucD, succinyl-CoA synthetase subunit alpha; TCA, tricarboxylic acid cycle.

**Figure 1:**
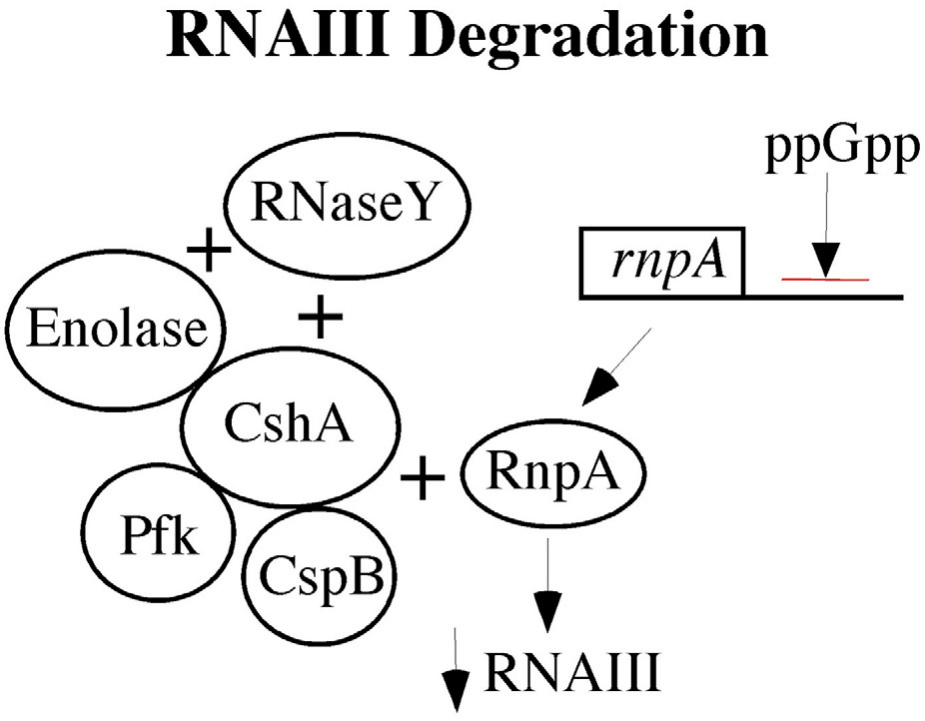
**The RNA degrasome is shown with respect to its impact on RNAIII as low levels of RNAIII are associated with persistent *S. aureus infections.*** An ATP-dependent *S. aureus* hellicase (CshA) is shown at the center with three proteins associated with it: Enolase, phosphofructokinase (Pfk), and cold shock protein B (CspB). Two RNase are shown, RNaseY and RnpA. RnpA has activity against RNAIII and its gene is negatively regulated by the alarmone, ppGpp.

Low levels of RNAIII can occur by two mechanisms. The *agr* quorum-sensing operon may produce less RNAIII or there may be increased RNAIII degradation to account for low levels of RNAIII. As noted above, the low RNAIII levels in electron transport type of SCVs are more likely related to increased RNAIII degradation. The RNA degrasome in *S. aureus* has recently been elucidated (Roux et al., 2011; Marincola et al., 2012; Redder and Linder, 2012; Oun et al., 2013), and a number of degrasome proteins are increased in SCVs as suggested by transcriptomics and proteomics data from *hemB* and *menD* mutant SCVs (Seggewiss et al., 2006; Kriegeskorte et al., 2011). A model of the RNA degrasome is provided in Figure 1.

The *hemB* mutant SCV has increased enolase, helicase (CshA), RsaA (a small noncoding RNA involved in stress responses and biofilm formation), *cspB* (cold shock protein B), SigB, SarA, and FruR (Seggewiss et al., 2006; Kriegeskorte et al., 2011). Of note, metabolism is embedded in the degrasome with enolase and phosphofructokinase (Pfk) directly interacting with CshA, but the effects that they have on CshA has yet to be characterized. Some of the targets of the degrasome are small nonprotein coding RNAs (sRNAs) that control central carbon metabolism (Geissmann et al., 2009; Bohn et al., 2010; Felden et al., 2011; Guillet et al., 2013; Romilly et al., 2014; Xue et al., 2014), and SCVs show changes in the levels of these sRNAs (Abu-Qatousch et al., 2010). This is not surprising as SCVs showed markedly increased levels of SigB (Mitchell et al., 2013), which positively regulates RsaA (Felden et al., 2011), and decreased levels of RsaE that regulates central carbon metabolism. The low levels of RsaE in SCVs are likely due to low levels of RNAIII, which is positively RsaE (Donegan and Cheung, 2009; Donegan et al., 2010; Guillet et al., 2013). In *Bacillus subtilis*, CspB associates with CshA (Hunger et al., 2006). CspB is included in the degrasome, and it is increased in *hemB* mutant SCVs (Seggewiss et al., 2006; Kriegeskorte et al., 2011). RNase Y processes *saeRS* mRNA so that it is active. (Marincola et al., 2012). Thus, as changes in RNA processing impact virulence factor production, TCA cycle, and biofilms, a number of the phenotypic characteristics of SCVs may be attributable to RNA processing.

A mutation in the ATP-dependent hellicase, *cshA*, in *S. aureus* results in a temperature-dependent SCV, with reduced growth at 30°C and complete growth inhibition at 22°C (Oun et al., 2013). Disruption of *cshA* results in higher RNAIII stability with increased hemolysis and reduced biofilm formation. Knocking *agrA* reversed the phenotype, indicating that *cshA* is genetically upstream of *agr* (Oun et al., 2013). In electron transport deficient SCVs, the higher levels of CshA might be expected to have a role in the lower levels of RNAIII.

As noted above, another potential element of the degradosome may be CspB. Of interest, a mutation in *cspB* in *S. aureus* also produces an SCV with reduced pigment production and increased resistance to aminoglycosides as found in electron transport SCVs (Duval et al., 2010). Other characteristics of SCVs such as RNAIII levels and expression of adhesins has not been studied. Similarly, the ability of the *cspB* mutant to persist has also not been reported.

## Small RNAs and SCVs

Based on investigations of cDNA libraries, phenotype-specific expression of sRNAs have been described for *S. aureus* (Abu-Qatousch et al., 2010). Selected sRNAs have also been found to in the *hemB* mutant SCV as compared to the wild type strain (Abu-Qatousch et al., 2010). Over expression of RsaE give SCVs that have decreased TCA cycle activity (Geissmann et al., 2009; Bohn et al., 2010). RsaE pairs with mRNAs of *citB*, *citZ*, and *sucCD*, thereby inhibiting their translation and reducing TCA cycle. While detailed metabolic studies are not available, one would anticipate that these organisms would have reduced electron transport and behave like electron transport deficient variants. Mutation of a sRNA, *rsaA*, has been show to alter virulence and cause persistent infection (Romilly et al., 2014). RsaA binds to MgrA, a global transcriptional regulator, and attenuates the severity of infections while increasing biofilm formation. Hence, it contributes to chronic infections (Romilly et al., 2014).

The higher levels of RsaA seen in electron transport SCVs might help to explain the increased biofilm formation in these variants as RsaA represses the synthesis of MgrA thereby increasing biofilm (Seggewiss et al., 2006). Also, SCVs have been reported to be readily killed by phagocytes (Quie, 1969), and this is also true of strains over-expressing RsaA (Seggewiss et al., 2006). Hence, increased amounts of RsaA may be contributing to the SCV phenotype. (A more detailed review of sRNAs in *S. aureus* is in Felden et al. (2011).

## Toxin-antitoxin, MazEF, and SCVs

**Figure 2:**
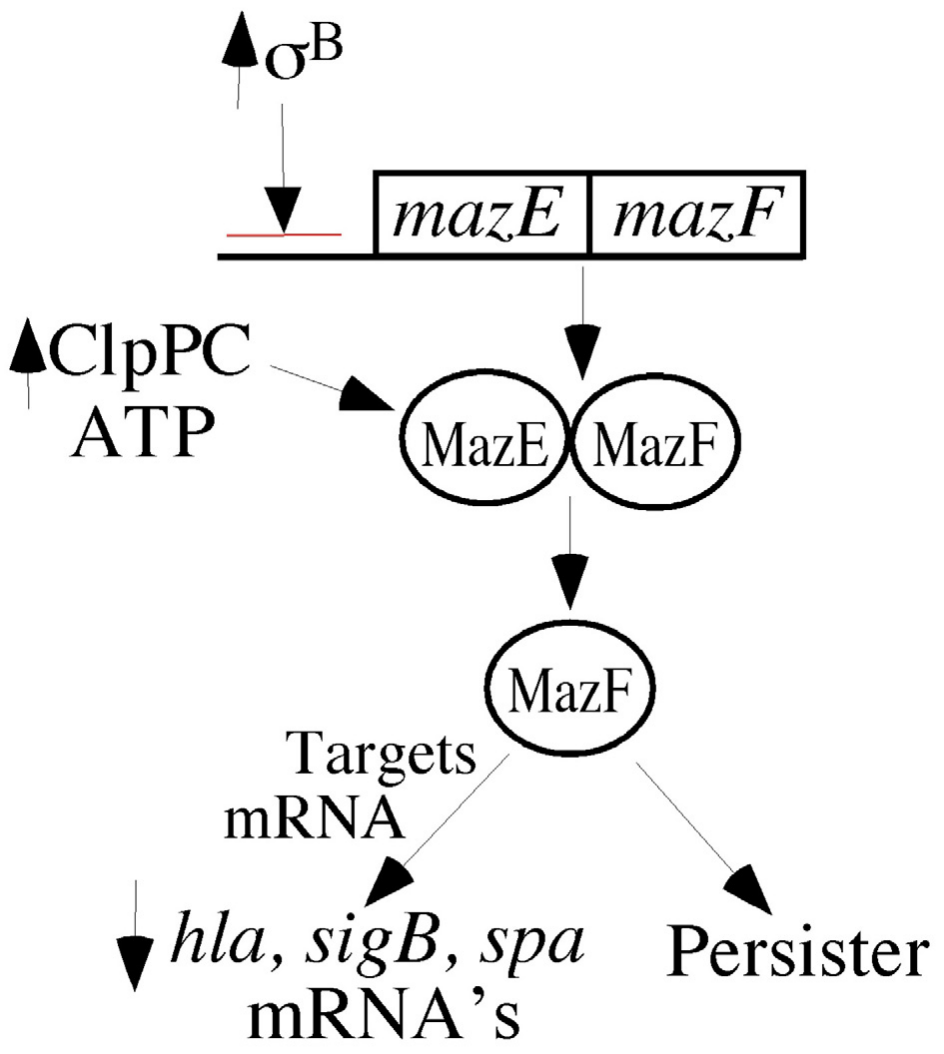
**MazEF are transcripbed as a pair of proteins, and their transcription is negatively regulated by SigmaB**. MazE is an anti-toxin protein that binds to MazF, thereby preventing its activity. MazF has RNase activity, which targets selected mRNAs in *S. aureus*. The ATP-dependent protease pair, ClpPC, digests the MazE antitoxin, thereby releasing MazF. Increased MazF has been associated with persistence.

Another system that is involved in mRNA stability in *S. aureus* is the MazEF toxin-antitoxin system (Fu et al., 2007, 2009; Donegan and Cheung, 2009; Donegan et al., 2010; Figure 2). Toxin-antitoxin systems are produced by many bacteria (Magnuson, 2007) and transcribed as an operon so that the toxin (MazF) is readily bond and neutralized by the antitoxin (MazE) (Fu et al., 2007, 2009; Donegan and Cheung, 2009; Donegan et al., 2010). MazE is an RNase that acts upon selected mRNAs. In *S. aureus*, MazE targets *sigB, hla, spa* mRNAs, but it avoids mRNAs from *recA, gyrB, sarA* (Fu et al., 2007, 2009). In most bacteria, the MazEF system is regulated by feedback inhibition by its own gene products, but in *S. aureus*, the negative regulation comes via SigB (Donegan and Cheung, 2009). The *mazEF* promoter allows transcription of not only *mazEF* but also the *sigB* operon, and activation of the *mazEF* promoter is needed for full SigB activity.

High levels of free MazF result in slow growth (SCVs), decreased pigment, and persistence, which can be achieved by overexpression of MazF or increased activity of ATP-dependent protease system, ClpCP, which proteolytically targets the anti-toxin, MazE (Fu et al., 2007, 2009; Donegan and Cheung, 2009; Donegan et al., 2010). This is compatible with the concept that toxin-antitoxin systems are involved in persistence (Magnuson, 2007). On the other hand, the low levels of ATP and ClpP and the higher levels σ^B^ in electron transport SCVs suggest that the MazEF system is probably not contributing to persistence in electron transport SCVs as electron transport SCVs have high levels of SigB (Mitchell et al., 2013).

## Mutation in *rpLF* and SCVs

The mutations in *rlf*, which encodes ribosomal protein L6, cause SCVs that have decreased pigmentation, like electron transport type of SCVs, and that allow for persistence (Norström et al., 2007; Lannergård et al., 2011). L6 is required for efficient protein synthesis, thus it is not surprising that cells with defective L6 would grow slowly. Rfl is also called FusE, which comes from the fact that these mutants are resistant to fusidic acid because of changes in ribosomal protein L6. In addition, *rfl* mutation in *E. coli* results in increased membrane fluidity and aminoglycoside resistance (Bosl and Böck, 1981), which is also seen with electron transport deficient SCVs. Aminoglycosides can also select for simultaneous fusidic acid resistance (Norström et al., 2007; Lannergård et al., 2011). Of note, in *S. aureus*, L6 is reduced in *hemB* mutants and under anaerobic growth (Fuchs et al., 2007; Kriegeskorte et al., 2011), suggesting that part of the phenotype seen in electron transport SCVs may relate to changes in L6. Little more in terms of the phenotype is known except that many, but not all of the *rfl* mutants, also carry mutations in *hem* or *men (gerC)*. These would be double SCVs as a mutation in *rlf* alone is sufficient to produce an SCV phenotype. Again, we see the ribosome, hence RNA, being involved in persistence and the SCV phenotype.

## Stringent response, persistence, and SCVs

When investigating a *S. aureus* strain from a patient with persistent and recurrent infection, a constitutively active mutation in *rsh*, a RelA homolog in *S. aureus*, was recovered (Gao et al., 2010). The mutation resulted defective ppGpp hypdrolase activity, thereby giving high levels of ppGpp and a persistent stringent response (Gao et al., 2010). Resistance to linezolid was also found. As ppGpp is the effector molecule in the stringent response (Geiger et al., 2012, 2014; Geiger and Wolz, 2014), which limits protein synthesis, it is not surprising that there is a growth defect, hence small colonies, i.e., an SCV (Geiger et al., 2012, 2014; Geiger and Wolz, 2014). Mutations in *rsh* result in reduced virulence (Geiger et al., 2012, 2014; Geiger and Wolz, 2014). Aside from the small colony size, the *rsh* mutant does not resemble the electron transport SCVs because *rsh* mutants show increased RNAIII and all of the characteristics seen when the Agr regulon is active.

## Arginine deiminase and persistence

In electron transport deficient SCVs, the *agrABDC* pathway, which is responsible for arginine deiminase activity, is markedly increased aerobically, whereas in the parent strains it is expressed only anaerobically (Kohler et al., 2003, 2008; Seggewiss et al., 2006; Makhlin et al., 2007). The anaerobic regulator of this operon, ArgR, is also significantly increased in the *hemB* mutant (Seggewiss et al., 2006), and it also regulates other normally anaerobic genes (Makhlin et al., 2007). This is of interest because the arginine catabolic mobile element (ACME) in USA300 is found along with the SCC*mec* element and is expressed aerobically whereas the other arginine deiminase operon are expressed only anaerobically (Thurlow et al., 2013). *S. aureus* USA300 is the hypervirulent epidemic strain in the USA. Arginine metabolism by ACME allows *S. aureus* to survive on the acidic environment of skin and within skin abscesses because it neutralizes acid with ammonia released during arginine catabolism (Thurlow et al., 2013). This provides a mechanism for the epidemiological observation that USA300 has an increased propensity to colonize the acidic environment of the skin chronically, which can be considered a type of persistence (Miko et al., 2012). Within ACME is another gene, *speG*, which encodes, spermidine acetyltransferase, a polyamine-resistance enzyme, thereby allowing *S. aureus* to survive spermidine exposure on the skin and in abscesses (Thurlow et al., 2013). Spermidine is otherwise a toxic product of arginine metabolism (Thurlow et al., 2013). The elucidation of the role of ACME in USA300 helps in the understanding how electron transport SCVs are able to survive for longer periods of time than other strains of *S. aureus* by using similar metabolic mechanisms.

## Metabolism and antibiotic-persisters

A large literature is available linking slow growth and resistance to antibiotics (Wood et al., 2013), and this is also found in *S. aureus* (Lechner et al., 2012). Discussing this type of persistence is beyond the scope of this review; however, one specific gene will be covered as it has a special link between phosphate metabolism and slow growth, hence an SCV, specifically *phoU*. PhoU is a global negative regulator that regulates genes involved in central carbon metabolism and cytochrome expression, thus it provides strong links to the electron transport type of SCVs (Li and Zhang, 2007; Gardner et al., 2014). In *S. aureus*, *phoU* can be found to be important for resistance to cationic antimicrobial peptides (Overton et al., 2011), which may relate to expression of the *dlt* and *snoD* operons that have been related to cationic antimicrobial peptide resistance (Peschel et al., 1999; Bayer et al., 2006). Both the *dlt* and *snoD* operons are regulated by PhoU. We have found that the electron transport type of SCVs are also persist in the presence of antibiotics and more resistant to cationic peptides (Koo et al., 1996; Gläser et al., 2014). Studies of *S. aureus phoU* mutants ability to persist within the host are not available, but one would surmise that this may be the case.

## Conclusions

Persistence and metabolism are intimately intertwined (Kahl, 2014), and it can be achieved via altering many pathways. Slow growth is common theme for persisters. The electron transport deficient SCVs show changes in many of the other pathways used for persistence, hence, the studies of these SCVs have a broader relevance for persistence. Of interest, altered RNA processing is suggested in the electron transport SCVs by the exceptionally low levels of RNAIII. A number of other persister types also show changes in RNA metabolism either directly through the RNA degrasome or at the ribosome. While the loss of *agr* is associated strongly with persistent and chronic infections, it is not associated with a growth defect. Naturally, there is a clear RNA effect in *agr* mutants in that the effector molecule, RNAIII, is not produced. Thus, there are many connections between the SCV phenotype and altered mRNA levels. There are also a number of shared phenotypic characteristics PhoU mediated changes and SCVs, but direct connections have yet to be studied. Finally, a growth defect is seen with high ppGpp levels, but these strains need to be tested for persistence.

### Conflict of interest statement

The authors declare that the research was conducted in the absence of any commercial or financial relationships that could be construed as a potential conflict of interest.
